# Supplementary material for: Structure of MHC class I-like MILL2 reveals heparan-sulfate binding and interdomain flexibility
Source: Nat Commun. 2018 Oct 18;9:4330. doi: 10.1038/s41467-018-06797-8 (PMC6193965; doi:10.1038/s41467-018-06797-8)
Supplement: Supplementary file 1 — Supplementary Information [file 41467_2018_6797_MOESM1_ESM.pdf]

Supplementary information

Structure of MHC class I-like MILL2 reveals heparan-sulfate binding and interdomain flexibility

Kajikawa et al.

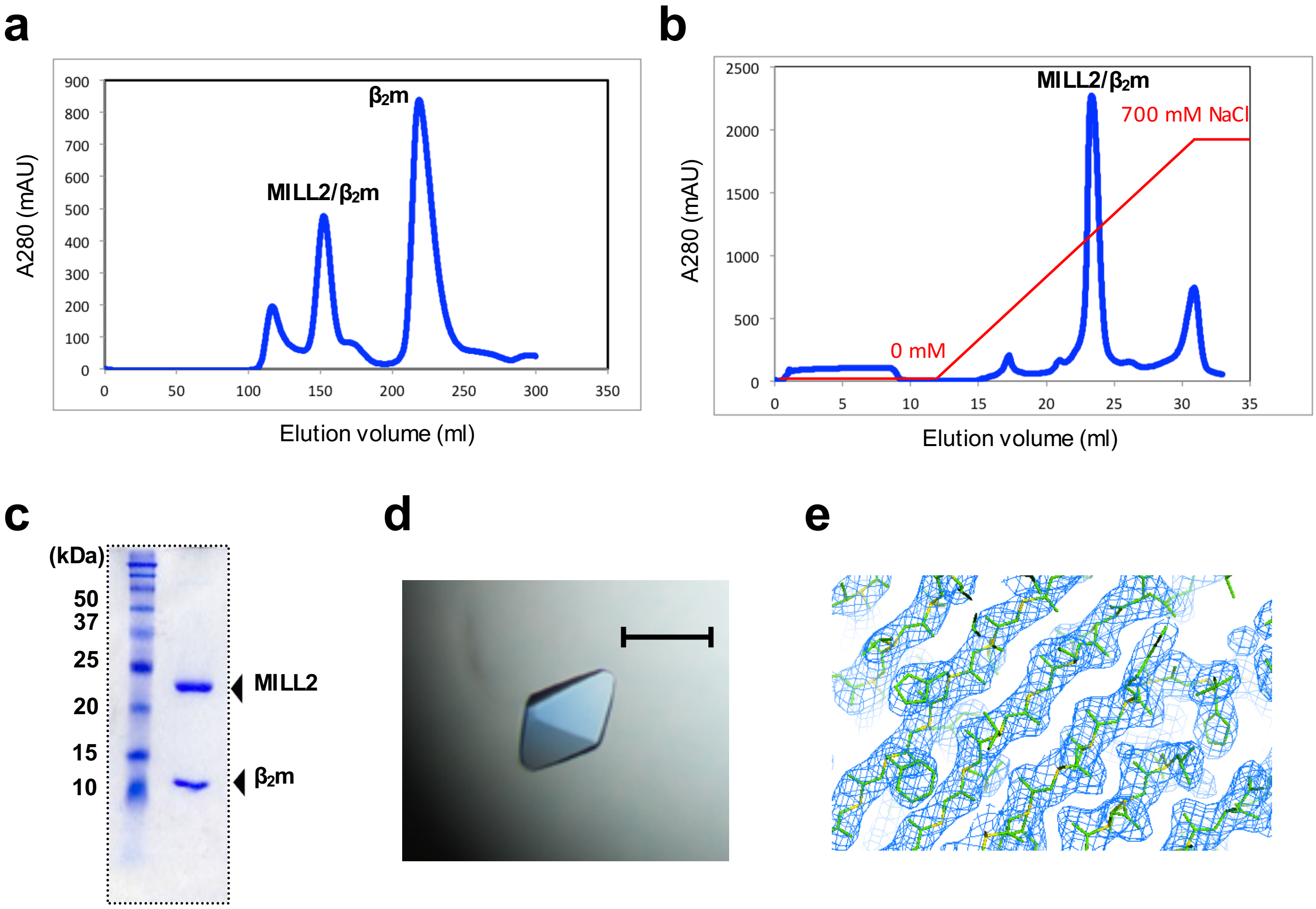

Supplementary Figure 1: Expression, purification and crystallization of MILL2.

(a) Size-exclusion chromatography of refolded MILL2 protein (MILL2/β_2m heterodimer). (b) Cation-exchange chromatography of MILL2/β_2m heterodimer. (c) SDS-PAGE analysis of the purified MILL2/β_2m heterodimer. (d) A crystal of MILL2/β_2m heterodimer grown by sitting-drop vapor diffusion. The scale bar represents 100 μm. (e) The MILL2/β_2m heterodimer 2*Fo*-*Fc* electron density map (1.0 sigma).

a

b

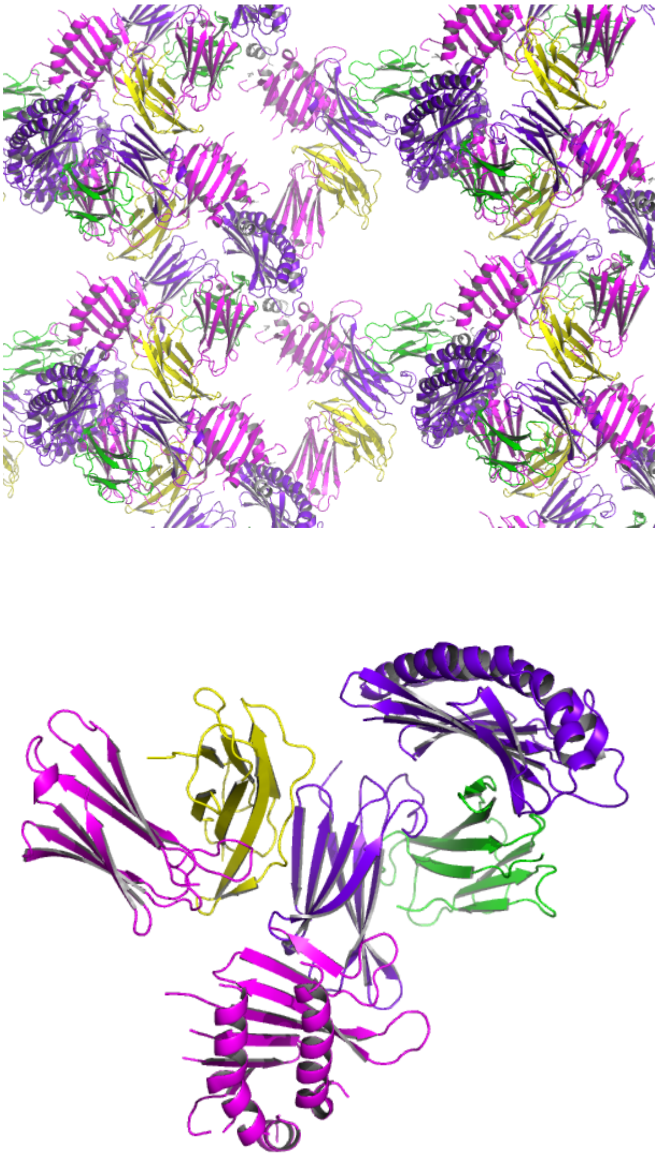

Supplementary Figure 2: The two conformations of MILL2 observed in crystals.

(a) Arrangement of MILL2/β_2m heterodimers in crystals and (b) a single asymmetrical crystal unit represented by ribbon diagrams. Purple, closed conformation of MILL2; magenta, open conformation of MILL2; green, β_2m associated with the closed conformation of MILL2; yellow, β_2m associated with the open conformation of MILL2.

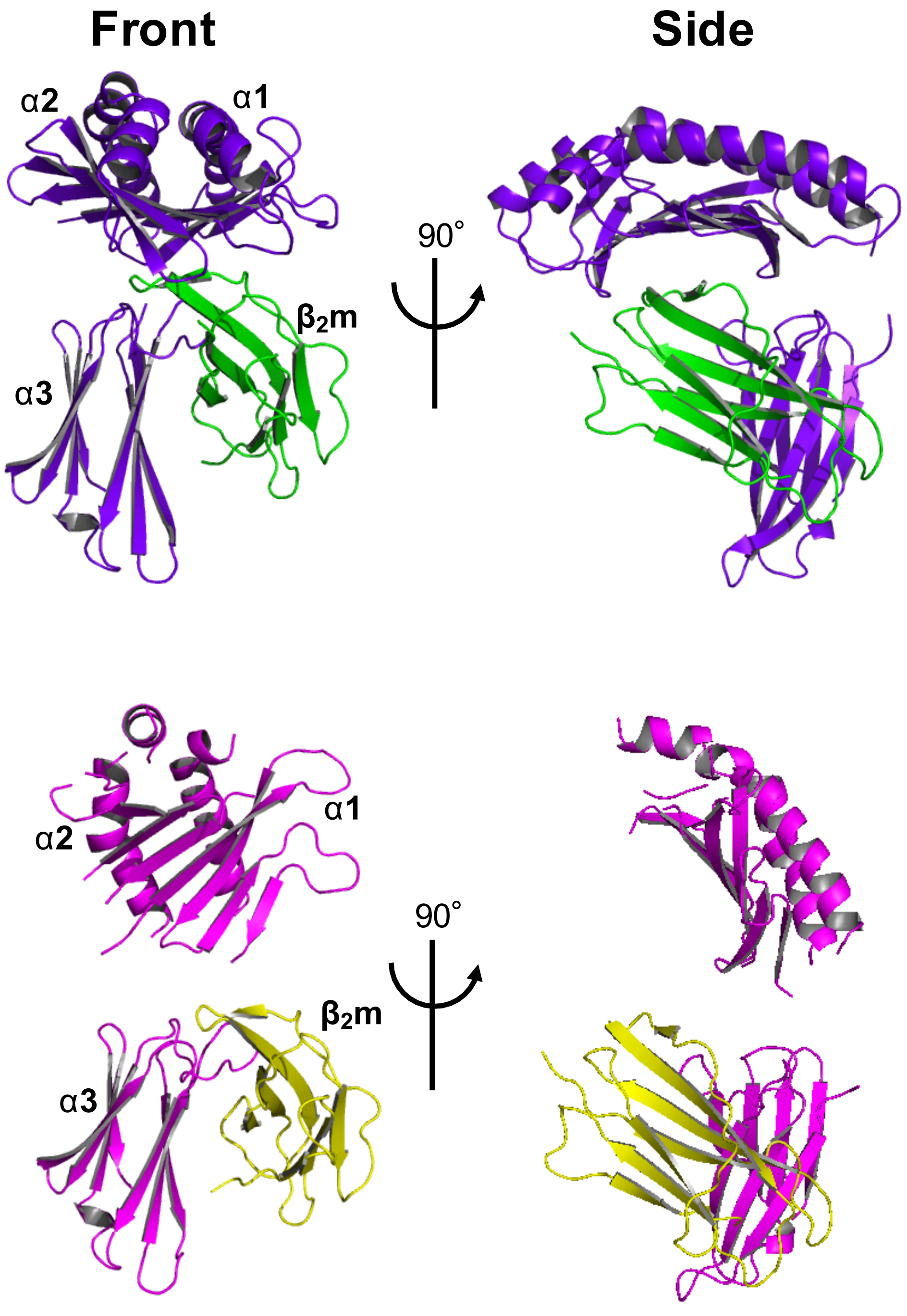

Supplementary Figure 3: Overview of MILL2 structure.

Front and side views of MILL2 structure represented by ribbon diagrams. Purple, closed conformation of MILL2 (α1, α2, and α3); green, β_2m associated with the closed conformation. Magenta, open conformation of MILL2 (α1, α2, and α3); yellow, β_2m associated with the open conformation. Two orientations are shown related by a vertical rotation of 90° .

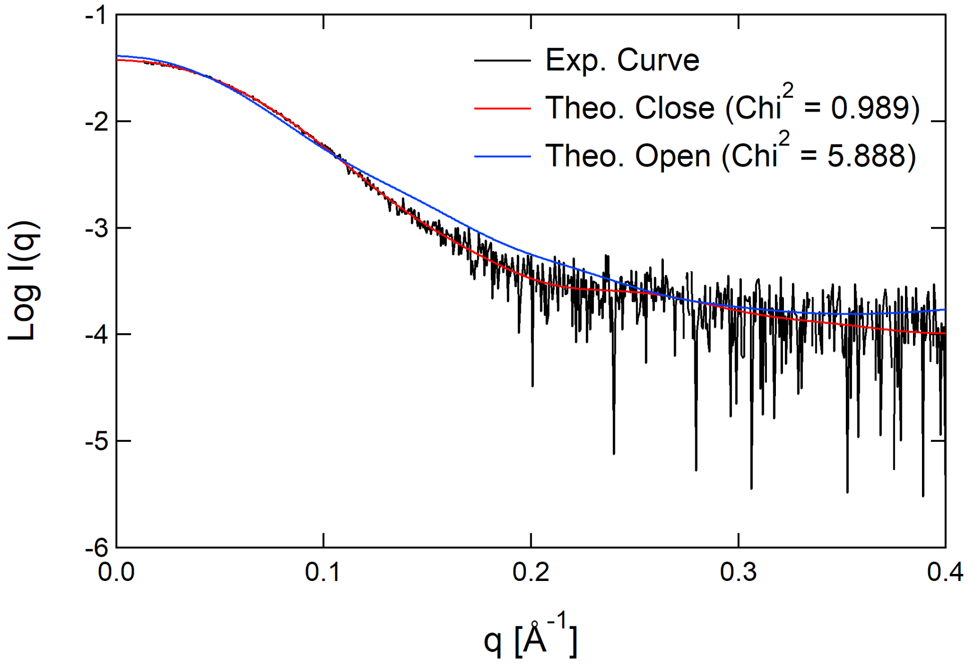

Supplementary Figure 4: SAXS profiles of MILL2.

The black line shows the experimental scattering profile. Red and blue lines are theoretical scattering profiles calculated from the close and the open conformation of PDBs respectively. Chi-square values indicate the coincidence with the experimental scattering are 0.989 for the closed conformation and 5.888 for the open conformation, respectively.

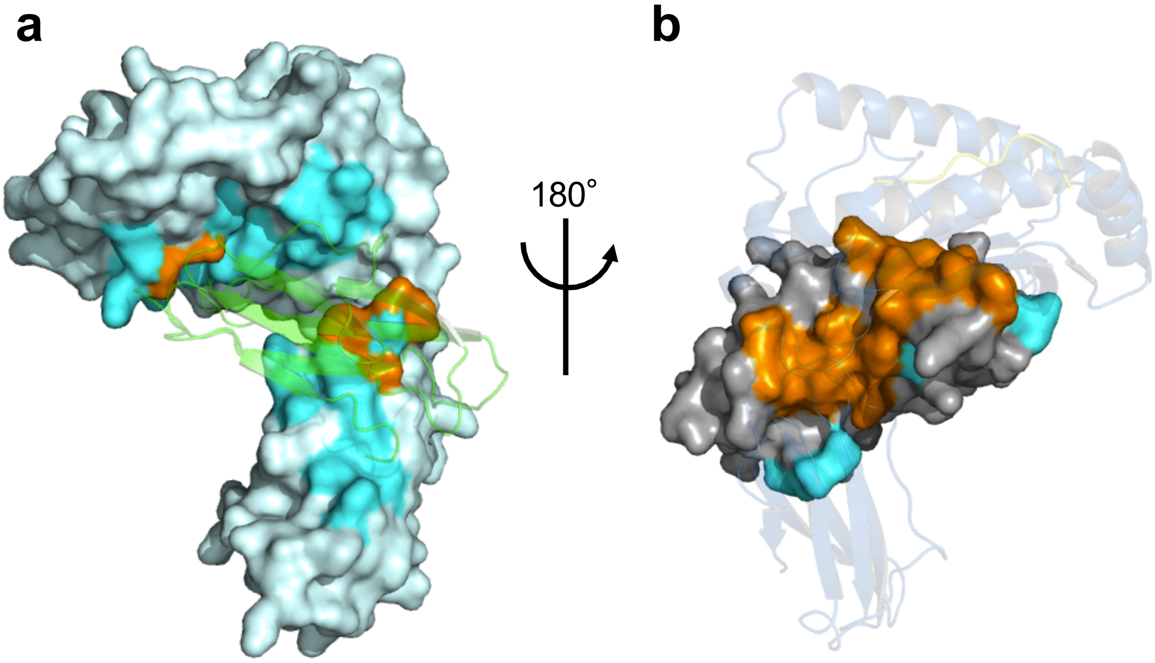

Supplementary Figure 5: Multiple contact sites with β_2m and MHC-I are not conserved in MILL2.

(a) Surface model of H-2D^b (PDB ID: 1CE6) showing contact residues with β_2m. Orange, conserved contact residues on H-2D^b between MILL2 and H-2D^b; cyan, H-2D^b-specific contact residues with β_2m. β_2m is represented by a transparent ribbon diagram (green). (b) Surface model showing contact residues on β_2m with H-2D^b. Orange, contact residues on β_2m conserved between MILL2 and H-2D^b; cyan, H-2D^b-specific contact residues on β_2m. H-2D^b heavy chains are represented by a ribbon diagram (skyblue).

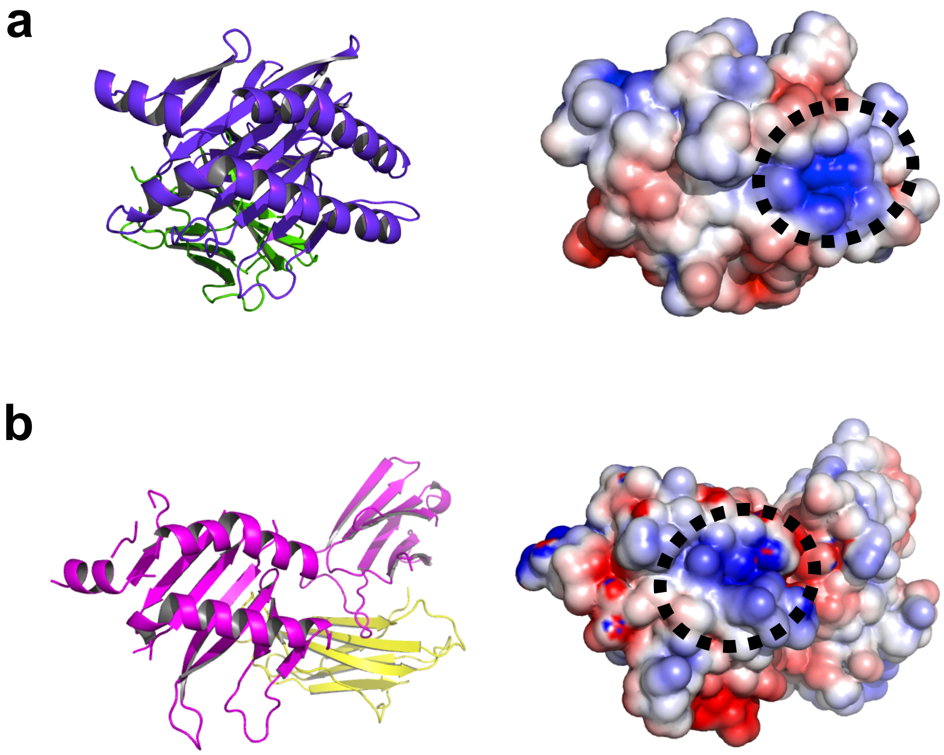

Supplementary Figure 6: Electrostatic surface potentials on the α1-α2 domains of MILL2.

(a) Top view of the closed or (b) open conformations of MILL2 shown by ribbon diagrams (left) and electrostatic surface potential models (right). Purple, closed conformation of MILL2; magenta, open conformation of MILL2; green, β_2m associated with the closed conformation of MILL2; yellow, β_2m associated with the open conformation of MILL2, respectively. Red and blue in the electrostatic surface potential models indicate negatively and positively charged areas, respectively. Dotted circles indicate the position of the basic patch on the α1-α2 domains of MILL2.

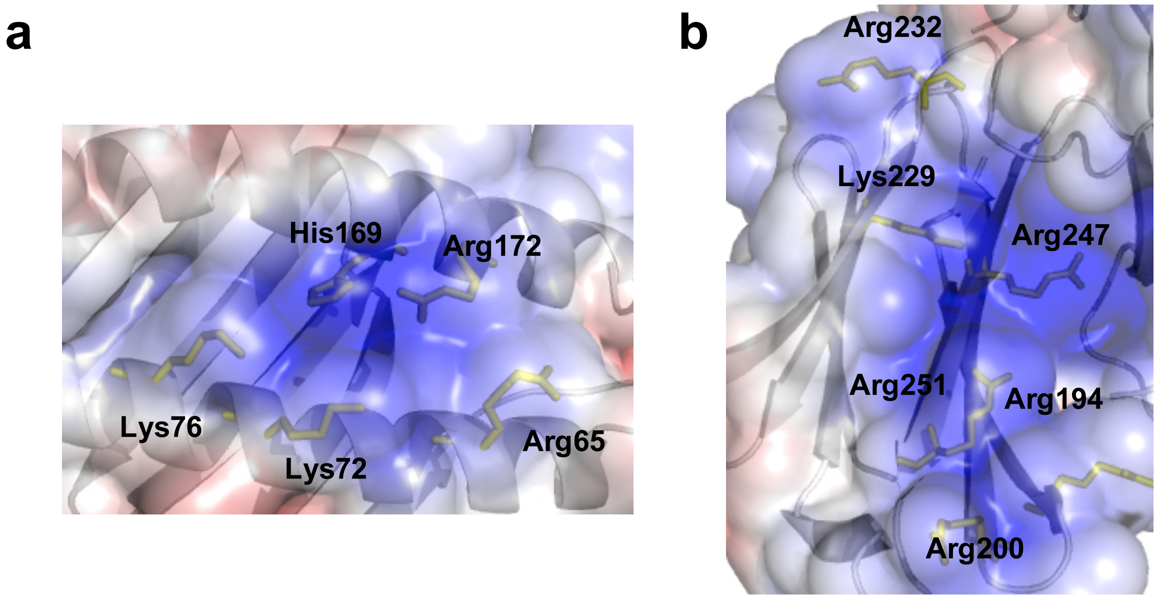

Supplementary Figure 7: The two basic patches on MILL2.

(a) The basic patch area on the α1-α2 domains represented by a transparent surface and ribbon diagram. Residues forming the basic charged surface are represented as sticks annotated with the corresponding amino acid number. (b) The basic patch area on the α3 domain shown as a transparent surface and ribbon diagram. The residues forming the basic charged surface are also shown as sticks annotated with the respective amino acid numbers.

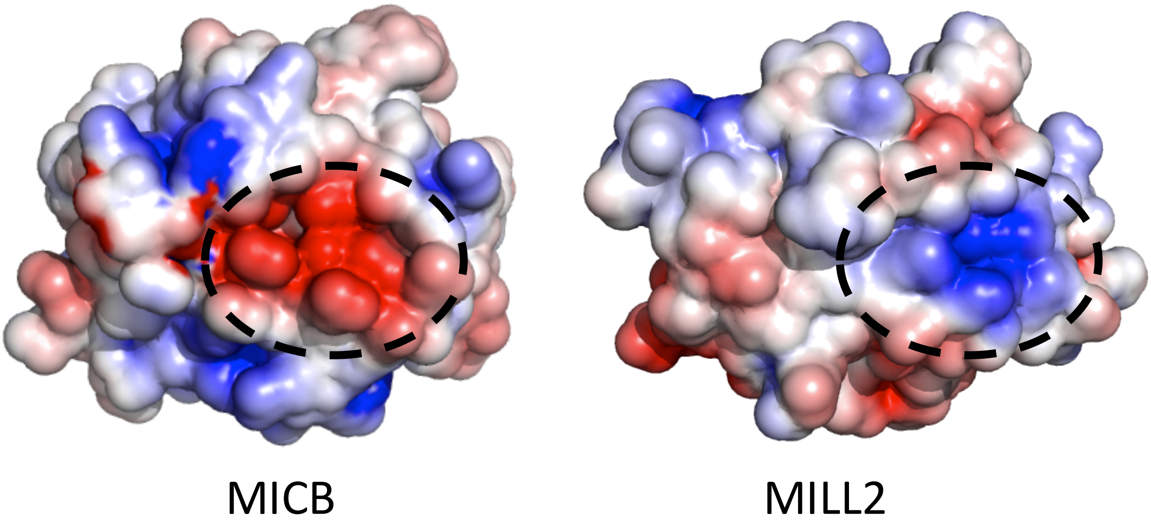

Supplementary Figure 8: Comparison of electrostatic surface potentials on the α1-α2 domains of MICB and MILL2.

The α1-α2 domains of MICB (PDB ID: 1JE6) and MILL2 are shown in a surface electrostatic potential model. Red and blue indicate negatively and positively charged areas, respectively. Dotted circles indicate the position of an acidic patch on MICB and a basic patch on MILL2.

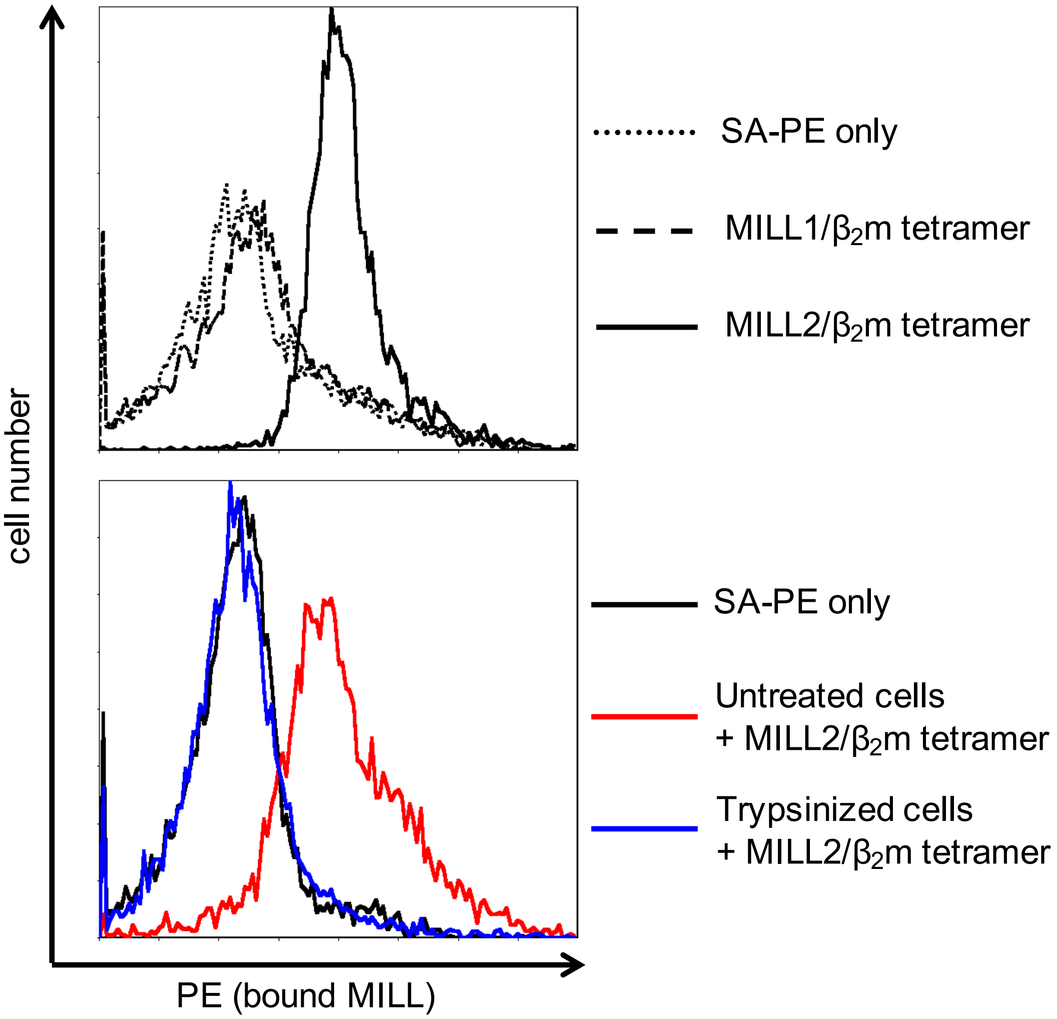

Supplementary Figure 9: MILL2 tetramer binds to the surface of NIH 3T3 cells.

NIH 3T3 cells were stained with MILL1/β_2m or MILL2/β_2m tetramer conjugated to phycoerythrin-labeled streptavidin (SA-PE) and analyzed by flow cytometry. Trypsinized cells were prepared by treatment with 0.05% trypsin at 37° C for 5 min.

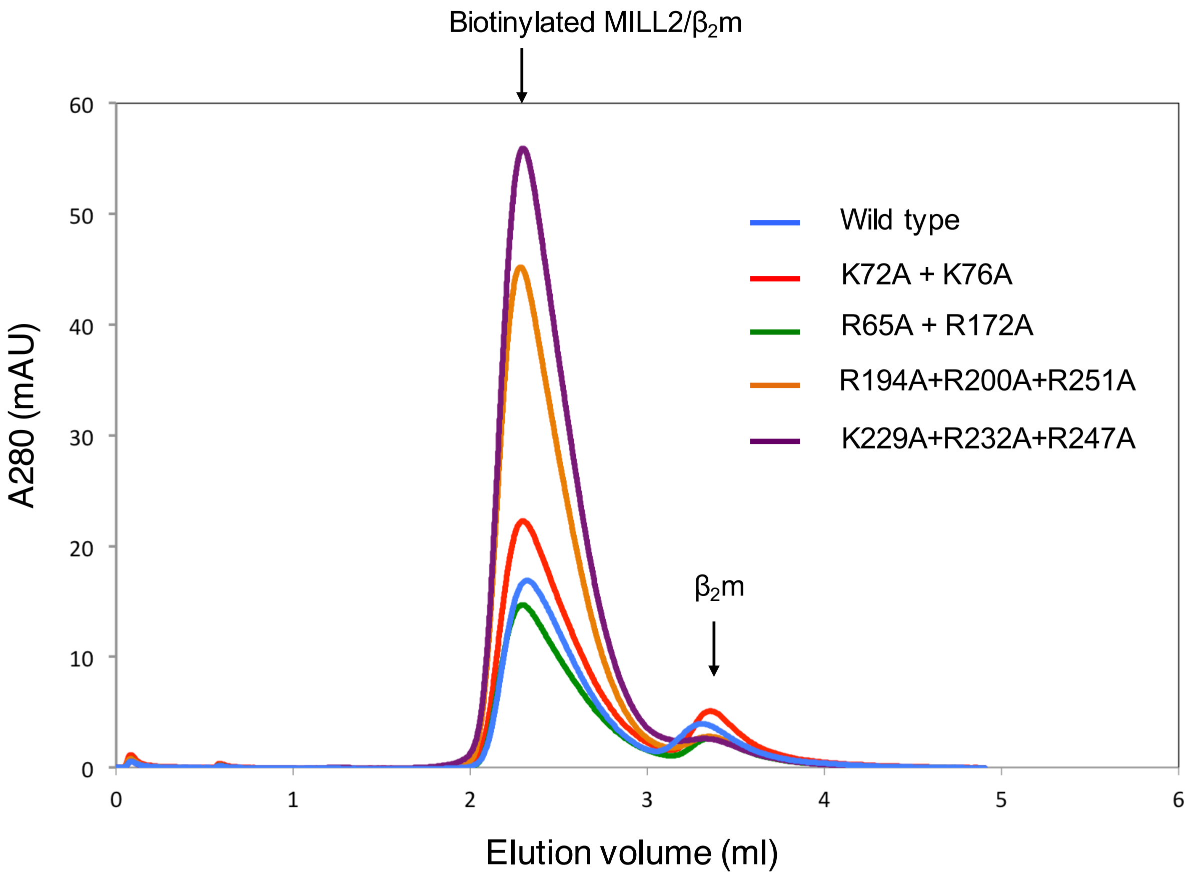

Supplementary Figure 10: Size exclusion chromatography of biotinylated MILL2/β_2m.

Size exclusion chromatogram of biotinylated MILL2 and alanine-substituted mutants of MILL2 are shown. MILL2/β_2m complexes were biotinylated with BirA enzyme. Following biotinylation and purification, complexes were analyzed on a Superdex™ 200 5/150 GL column (GE Healthcare Life Sciences) to confirm the structural stability of these mutants.

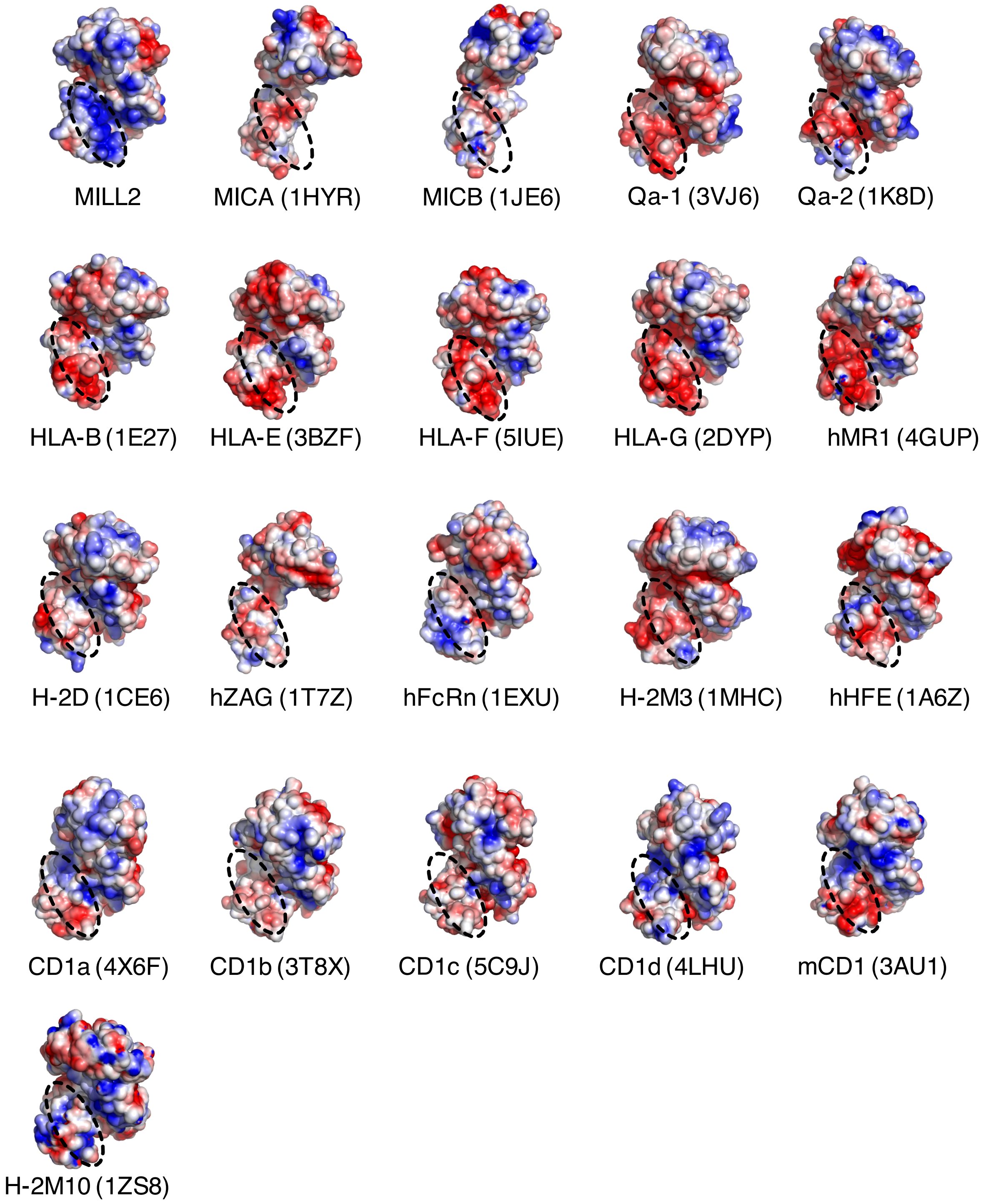

Supplementary Figure 11: Electrostatic analysis of MHC-I molecule family members.

Front views of different MHC-I molecules shown as electrostatic surface potential models. Red and blue indicate negatively and positively charged areas, respectively. Dotted circles indicate the basic patch on the α3 domain of MILL2 and the corresponding areas on the other MHC-I molecules. The four letter code in parentheses indicates the PDB ID.

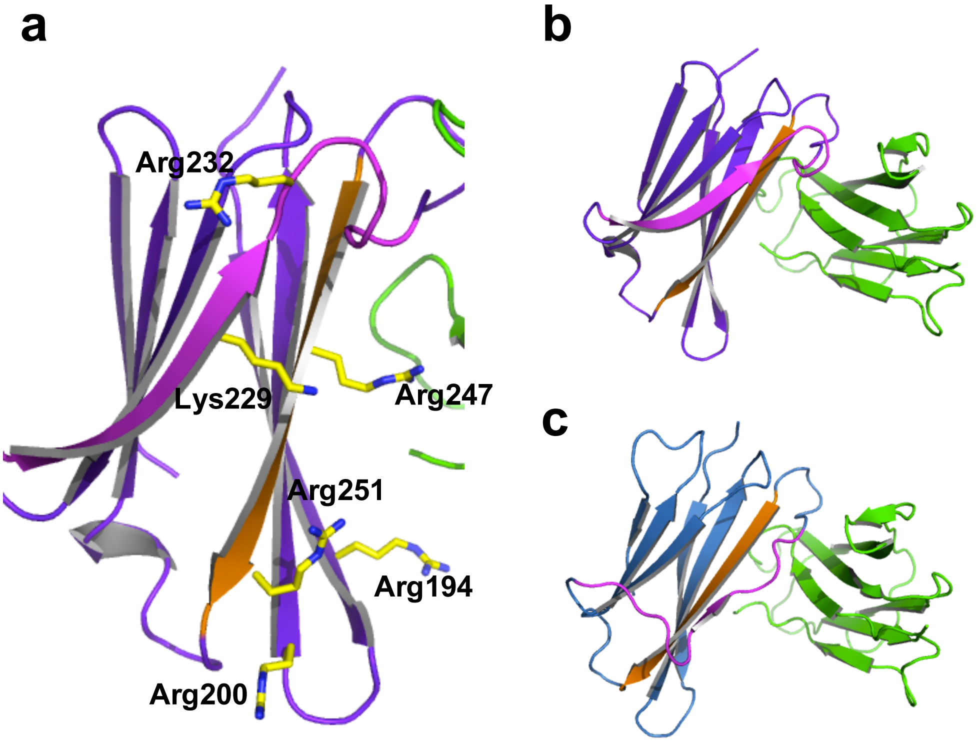

Supplementary Figure 12: The position of fourth β strand in the α3 domain of MILL2 is markedly different from other MHC-I molecules.

(a) A ribbon model showing the α3 domain of MILL2 (purple). The fourth and fifth β-strands of the α3 domain are represented in magenta and orange, respectively. The side chains of Arg194, Arg200, Lys229, Arg 232, Arg247 and Arg251 residues are represented by sticks. (b, c) Ribbon models showing the α3 domains of MILL2 (b) or H-2D^b (PDB ID: 1CE6) (c) with β_2m. Skyblue, H-2D^b; purple, MILL2; green, β_2m. The fourth and fifth β-strands in the α3 domain of MILL2 and the corresponding regions of H-2D^b are indicated in magenta and orange, respectively.

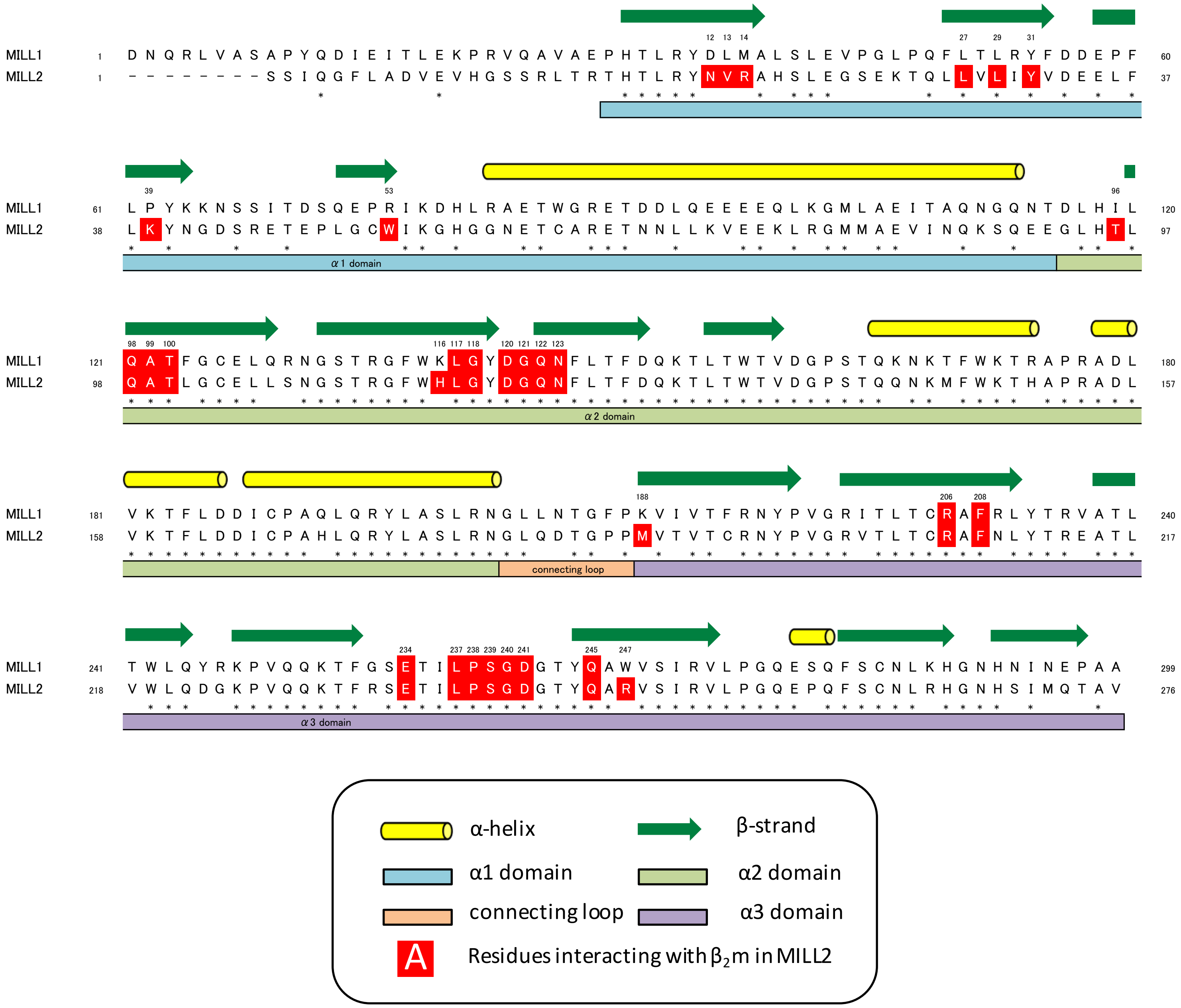

Supplementary Figure 13: Alignment of extracellular domain sequences of murine MILL1 and MILL2 showing contact residues with β_2m.

Alignment of the extracellular domain sequences of MILL1 and MILL2. Residues interacting with β_2m in MILL2 are highlighted by red with the amino acid number. Conserved β_2m-interacting residues in MILL1 with MILL2 are also highlighted in red. Conserved residues between MILL2 and MILL1 are indicated by an asterisk.

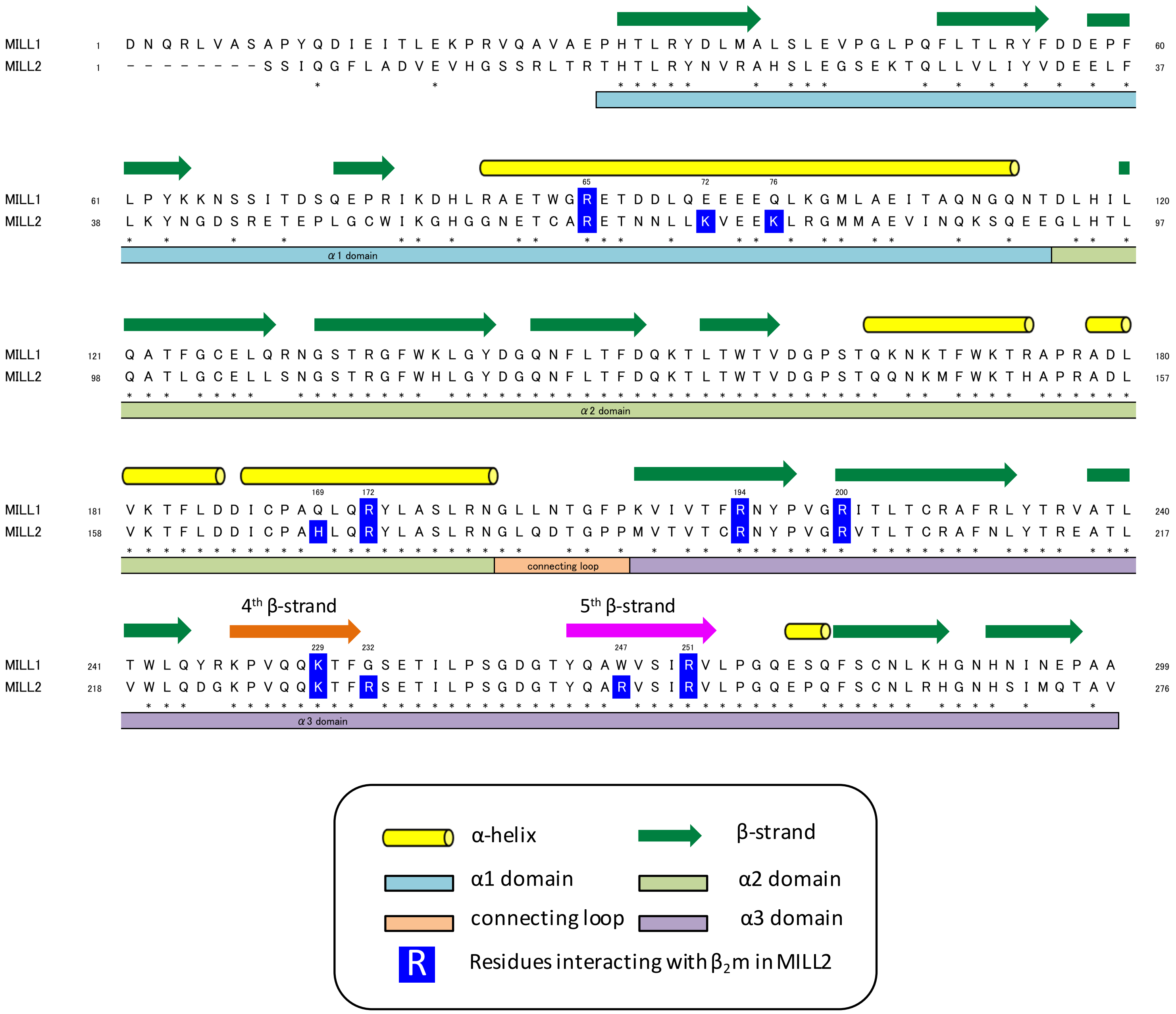

Supplementary Figure 14: Basic residues forming basic patches on α1-α2 domains and α3 domain.

Alignment of extracellular domain sequences between murine MILL1 and MILL2. Residues forming basic patches in MILL2 are highlighted blue with the amino acid number. Conserved residues in MILL1 with MILL2 residues forming basic patches are also highlighted in blue. Asterisk indicates conserved residues between MILL2 and MILL1.

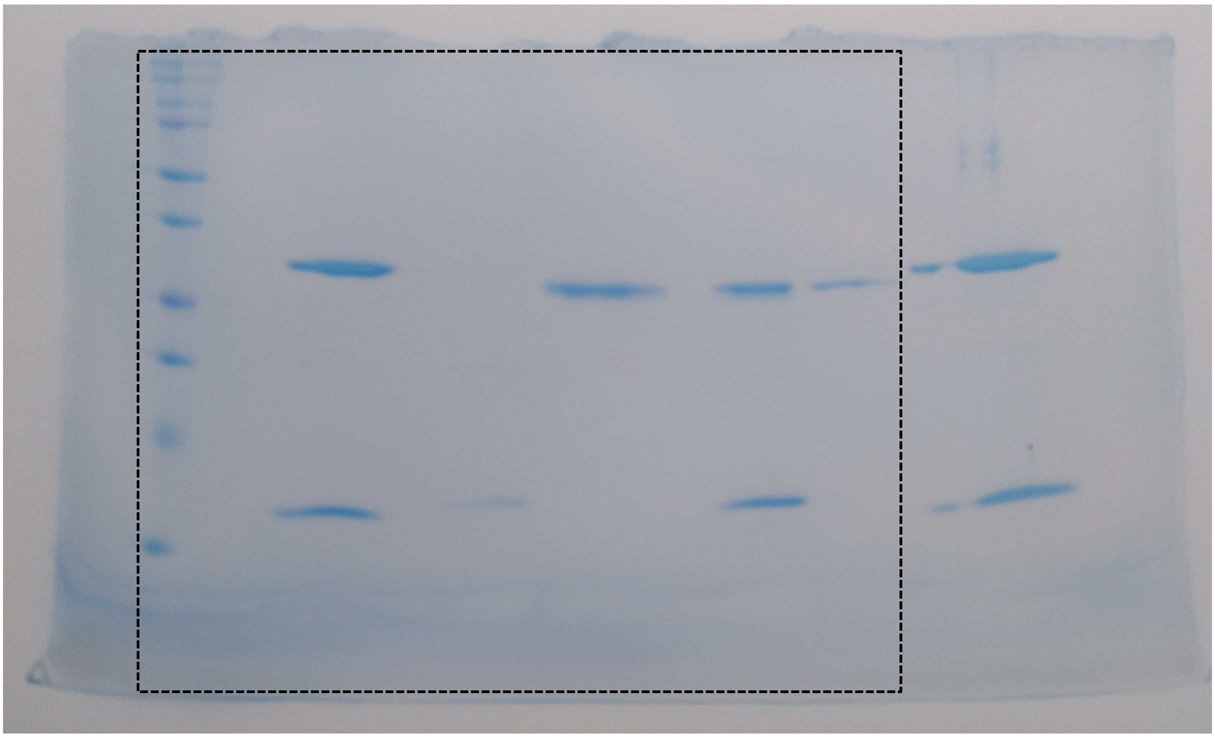

Supplementary Figure 15: Uncropped scanned image of the SDS-PAGE gel shown in Figure 4b.

Supplementary Table 1 : **Data collection and refinement statistics**

	MILL2
**Data collection**	
Space group	$P2_12_12_1$
Cell dimensions	
a, b, c (Å)	89.63, 93.50, 138.12
Resolution (Å)$^{\neq}$	50.00-2.15 (2.19-2.15)
R_{meas}	0.079 (1.313)
R_{pim}	0.032 (0.619)
$I / \sigma I$	36.4(1.8)
Completeness (%)	99.9 (100.0)
Multiplicity	7.3 (7.3)
$CC^1/_2{}^{\neq}$	(0.622)
**Refinement**	
Resolution (Å)$^{\neq}$	44.82-2.148 (2.225-2.148)
No. unique reflections	63883 (6278)
R_{work} / R_{free}	0.2155(0.742) / 0.2487(0.2915)
No. atoms	
Protein/Water	5392/204
Sulfate ion	5
Average B-factors (Å^2)	
Protein/Water	60.20/59.50
Sulfate ion	56.00
Wilson B factor (Å^2)	45.43
R.m.s. deviations	
Bond lengths (Å)	0.003
Bond angles (°)	0.82
Ramachandran plot	
Favored regions (%)	97.0
Outliers (%)	0.45

Supplementary Table 2 : SAS data collection and analysis.

**A. Sample details**	
Organism	*Mus. musculus*
Source	E.coli expressed
Extinction coefficient (A_{280nm}, Abs $_{0.1\%}$ (w/v))	1.3
Partial specific volume $\bar{\upsilon}$ ($cm^3 g^{-1}$)	0.733
Mean scattering contrast $\Delta\bar{\rho}$ (cm^{-2})	2.906×10^{10}
M from chemical composition (Da)	44688
Concentration, (mg ml^{-1})	0.89
Concentration values measured and method	UV-Visible spectroscopy
Solvent composition	10mM HEPES-NaOH, 150mM NaCl, pH7.4
**B. SAS data collection parameters**	
Source, instrument	Photon Factory, BL-10C
Wavelength (Å)	1.0
Camera Length (mm)	2009
Beam geometry (μm)	V350 × H550
	Bent cylindrical mirror + 2 slits + 1 pinhole
Q -measurement range ($Å^{-1}$)	0.00753-0.424
Absolute scaling method	Comparison with scattering from pure H_2O
Basis for normalization to constant counts	Normalized to incident intensity by μ ion chamber
Method for monitoring radiation damage	Data frame-by-frame comparison
Exposure time, number of exposures	20 sec, 296 images
Sample configuration including path length	H1.5 × W3.0 × t1.0 mm
Sample temperature (K)	293
**C. Software for SAS data analysis**	
SAS data processing, Basic analyses	*SAngler*, *CRYSOL*
Calculation of $\bar{\upsilon}$, $\Delta\bar{\rho}$ values	*MULCh*[1]
**D. Guinier analysis**	
Q range ($Å^{-1}$) ($Q\times R_g$ range)	0.0129 – 0.0549 (0.31 – 1.30)
I(0) (cm^{-1})	0.037+/- 0.0001
M from *I*(0)	55.2 kDa
R_g (Å)	23.7+/- 1.3
R_g (Å) from the close form (CRYSOL)	23.9
R_g (Å) from the open form (CRYSOL)	26.8

Reference

1. Whitten, A.E., Cai, S. & Trewhella, J. MULCh: Modules for the analysis of small-angle neutron contrast variation data from biomolecular assemblies. *J Appl Crystallogr* **41**, 222-226 (2008).

Supplementary Table 3: Primer sequences used in this study

Plasmid	Forward primer	Reverse primer
pGMT-MILL2	5'-TACTATTAATGTCTAGTATTCAAGGTACTCATACTTTACGCTATAATGTCAGAGCCCA-3'	5'-CCAAGCTTCTATTAGACAGCTGTCTGCATGATGC-3'
pGMT-β_2m	5'-TACTATTAATGATCCAGAAAACCCCTCAAATTC-3'	5'-ATGAAAGCTTCTATTACATGTCTCGATCCCAGTAGACG-3'
pGMT-MILL1-birA	5'-ACTATTAATGGACAACCAAAGACTGGT-3'	5'-ATGAGGATCCGGCAGCAGGTTCATTGATAT-3'
pGMT-MILL2-birA	5'-TACTATTAATGTCTAGTATTCAAGGTACTCATACTTTACGCTATAATGTCAGAGCCCA-3'	5'-ATGAGGATCCGACAGCTGTCTGCATGATGC-3'

* Underlines indicate restriction enzyme sites: ATTAAT (*Psh* BI), AAGCTT (*Hin* dIII), GGATCC (*Bam* HI)
