# Supplementary material for: Structure of MHC class I-like MILL2 reveals heparan-sulfate binding and interdomain flexibility
Source: Nat Commun. 2018 Oct 18;9:4330. doi: 10.1038/s41467-018-06797-8 (PMC6193965; doi:10.1038/s41467-018-06797-8)
Supplement: Supplementary file 3 — Description of Additional Supplementary Files [file 41467_2018_6797_MOESM3_ESM.pdf]

Description of Additional Supplementary Files

File Name: Supplementary Data 1
Description: Interchain contacts between heavy chains and β2m
